# Extensively drug-resistant ESBL-producing enterobacterales and coexisting antimicrobial resistance genes are associated with mortality in critically ill patients with cancer

**DOI:** 10.1038/s41598-026-67637-0

**Published:** 2026-09-02

**Authors:** Samah Mohammed Refay, Noha Gaber Sayed, Waleed M. El Kazzaz

**Affiliations:** 1 Botany and Microbiology Department, Faculty of Science, Suez Canal University, 4.5 Km Ring Road, Ismailia, 41522 Egypt; 2 Clinical Pathology Department, South Egypt Cancer Institute, Assiut University, El-Methak Street, Assiut, 71515 Egypt

**Keywords:** ESBL, *K. pneumoniae*, *E. coli*, Cancer, XDR, Cancer, Diseases, Microbiology

## Abstract

ESBL-producing bacteria are a problem in patients with cancer. The emergence of XDR phenotypes with ESBLs increases mortality. We aimed to evaluate ESBL-producing bacteria, the prevalence of MDR and XDR phenotypes, ESBL genes, and mortality predictors in patients with cancer in the ICU. Different clinical cultures were collected from patients in oncology ICUs. We used the VITEK2 system to identify isolates and assess antibiotic sensitivity, and the combination disk method to detect ESBL phenotypes. β-lactamase, ESBL, and carbapenem genes were detected using PCR. 136 isolates were ESBL-producing. Respiratory cultures were the main source, followed by blood cultures. *K. pneumoniae* was the most common, followed by *E. coli*. Isolates showed high resistance to third- and fourth-generation cephalosporins and carbapenem antibiotics. 84.6% and 13.2% of isolates were XDR and MDR, respectively. The most harbored genes were SHV and TEM (94.1%), CTXM-15 (59.6%), SHV-12 (67.6%), and NDM (78.7%). 38.2% of isolates harbored seven β-lactamase, ESBL, and carbapenem genes. *K. pneumoniae* significantly harbored more ESBL and carbapenem genes than *E. coli*. Patient age, relapses, and hospital stay are predictors of mortality in patients. An improved surveillance program and antibiotic stewardship are urgently needed.

**Supplementary Information:**

The online version contains supplementary material available at https://doi.org/10.1038/s41598-026-67637-0.

## Introduction

Multidrug-resistant (MDR) bacteria have become a global health problem, particularly *Enterobacterales* that are resistant to third-generation cephalosporins^1^. Bacteria resist β-lactam antibiotics through the primary mechanism of β-lactamases. Infection with MDR and extended-spectrum beta-lactamase (ESBL), associated with antibiotic resistance, fails antibiotic treatment, raising morbidity and mortality^2^.

Patients with hematological malignancies are vulnerable to infection due to chemotherapy and neutropenia, which causes immunosuppression, and are susceptible to ESBL-producing bacteria^3,4^. Antibiotic resistance can disrupt cancer treatment and increase treatment costs, particularly in countries with limited resources. Infection with multidrug-resistant gram-negative bacteria poses a challenge in cancer management and increases mortality and morbidity^5^. Patients in the intensive care unit (ICU) are exposed to ESBL-producing and multidrug-resistant bacteria, contributing to a global health problem^6^. 

Invasive infections caused by ESBL *Enterobacterales* (ESBL-PE) are a significant risk factor^7^. ESBL genes are prevalent among *E. coli* and *Klebsiella pneumoniae*, causing urinary tract and bloodstream infections, particularly in pediatric patients^8^. The main causes of blood infections in patients with hematological cancer are *Klebsiella spp.* and *E. coli*, whereas increased mortality is associated with carbapenem resistance, ESBL, and prolonged bacteremia^9^. *K. pneumoniae* that produces ESBL and methicillin-resistant *Staphylococcus aureus* (MRSA) are valued as top pathogen-drug combinations, ascribing mortality to antibiotic resistance^10^.

*K. pneumoniae* and *E. coli* producing ESBL and carbapenemases limit therapy options, which necessitate the use of last-resort antibiotics. In addition to chromosomal or plasmid-mediated resistance, AmpC presents resistance to ESBL, cephamycin, and a combination of β-lactam and β-lactam inhibitors, limiting choices in the ICU^11^. Over the past decade, the incidence of ESBL-producing bacteria has increased in hospital settings and the community because of the pandemic, with CTX-M being the major type of ESBL^2,12^. However, bla_CTX−M_ is the most widespread ESBL gene, and the prevalence of other significant ESBL genes is understudied, which can lead to worse clinical outcomes^13^.

The emergence of extensively drug-resistant (XDR) and pan-drug-resistant (PDR) bacterial strains is an alarming development in infectious diseases^14^. Bacteremia produced by carbapenem-resistant organisms and third-generation cephalosporin-resistant *Enterobacterales* aggravates therapy costs, hospital stay, morbidity, and mortality^15^.

We aimed to screen ICU cancer patients for ESBL, MDR, and XDR bacteria. To evaluate outcomes associated with ESBL infection and identify risk factors associated with mortality (the main outcome of ESBL infection) in cancer patients infected with ESBL bacteria.

## Results

### Demographic characteristics

A total of 201 pathogenic strains were isolated from different culture sources of cancer patients in the ICU. Among these, 58 (28.86%) were gram-positive, and 143 (71.14%) were gram-negative. A total of 136 (95.1%) of gram-negative bacteria were ESBL producers. The median age of the patients was 50 years (range, 4–79 years); 73(53.7%) patients were female; 88 (64.7%) had solid tumors, and 75 (55.1%) received chemotherapy (Table 1). Of the patients, 29.4% had comorbidities, and 70 (51.5%) had relapses. A total of 112 (82.4%) patients had a central venous catheter inserted. A total of 52 (38.2%) patients had previously used antibiotics.

**Table 1:** Patients’ demographic and clinical characteristics.

Characteristics		
Age	Median (Range)	50(4–79)
Age groups	Child	24(17.6%)
	Adult	112(82.4%)
Gender	Male	63(46.3%)
	Female	73(53.7%)
Malignancy type	Solid tumor	88(64.7%)
	Hematological malignancy	48(35.3%)
Treatment	Chemotherapy	75(55.1%)
	Radiation therapy	22(16.2%)
	Surgery	33(24.3%)
	No treatment	6(4.4%)
Comorbidity	Yes	40(29.4%)
Relapses	Yes	70(51.5%)
Mechanical ventilation	Yes	62(45.6%)
Blood CVC	Yes	112(82.4%)
Urinary catheter	Yes	113(83.1%)
Surgical drain	Yes	14(10.3%)
Tracheostomy tube	Yes	11(8.1%)
Surgery	Yes	41(30.1%)
Prior antibiotic use	Yes	52(38.2%)
Prior hospital admission	Yes	80(58.8%)
Prior ICU admission	Yes	36(26.5%)
Hospital stay period (days)		10(0–74%)

Data were expressed as median (range) and frequency (percentage). CVC: central venous catheter. ICU: Intensive Care Unit.

### Bacterial distribution

A total of 136 ESBL-positive isolates were obtained as follows: from respiratory cultures, 52 (38.2%), followed by blood cultures, 45 (33.1%), and urine cultures (17.6%) (Supplementary Figure S1). Table 2 shows that *K. pneumoniae* was the most prevalent (48.5%), followed by *E. coli* (16.2%), *Acinetobacter baumannii* (6.6%), and others with low percentages. *Klebsiella pneumoniae* was the most common among blood, respiratory, and other cultures, followed by *E. coli* in blood and other cultures, while *E. coli* was the most common in urine cultures (37.5%), followed by *K. pneumoniae* (29.2%).

**Table 2:** Distribution of ESBL-producing bacterial isolates across various culture sources and causative pathogens.

	Blood culture (*n* = 45)	Urine culture (*n* = 24)	Respiratory cultures (*n* = 52)	Other cultures (*n* = 15)	Total (*N* = 136)
Klebsiella pneumoniae	23(51.1%)	7(29.2%)	30(57.7%)	6(40%)	66(48.5%)
*Escherichia coli*	7(15.6%)	9(37.5%)	3(5.8%)	3(20%)	22(16.2%)
*Acinetobacter baumannii complex*	2(4.4%)	2(8.3%)	4(7.7%)	1(6.7%)	9(6.6%)
*Pseudomonas spp.*	1(2.2%)	3(12.5%)	1(1.9%)	1(6.7%)	6(4.4%)
*Serratia ssp.*	2(4.4%)	2(8.3%)	1(1.9%)	1(6.7%)	6(4.4%)
*Enterobacter cloacae complex*	3(6.7%)	0(0%)	2(3.8%)	0(0%)	5(3.7%)
*Raoultella spp.*	1(2.2%)	0(0%)	2(3.8%)	1(6.7%)	4(2.9%)
*Pantoea agglomerans*	0(0%)	1(4.2%)	3(5.8%)	0(0%)	4(2.9%)
*Klebsiella oxytoca*	1(2.2%)	0(0%)	2(3.8%)	0(0%)	3(2.2%)
*Klebsiella aerogenes*	0(0%)	0(0%)	0(0%)	3(20%)	3(2.2%)
*Burkholderia spp.*	3(6.7%)	0(0%)	0(0%)	0(0%)	3(2.2%)
*Sphingomonas paucimobilis*	2(4.4%)	0(0%)	0(0%)	0(0%)	2(1.5%)
*Citrobacter spp.*	0(0%)	0(0%)	3(5.8%)	0(0%)	3(2.2%)

Data were expressed as frequencies and percentages.

### Antibiotic resistance

#### General resistance and resistance patterns

All isolates expressed high resistance to ampicillin (97.1%) and cefazolin (97.8%), followed by ceftriaxone (96.2%) and ampicillin/sulbactam (92.9%), whereas gentamicin showed the lowest resistance (69.1%) (Table 3).

**Table 3:** Antibiotic susceptibility profile and resistance patterns for all isolated Gram-negative bacteria producing ESBL.

	Sensitive	Intermediate	Resistant
Penicillins			
Ampicillin	3(2.9%)		101(97.1%)
Ampicillin/Sulbactam	5(4.4%)	3(2.7%)	105(92.9%)
Piperacillin/Tazobactam	12(8.8%)	3(2.2%)	121(89%)
Cephalosporins			
First generation			
Cefazolin	2(1.5%)	1(0.7%)	133(97.8%)
Third generation:			
Ceftazidime	5(3.7%)	10(7.4%)	121(89%)
Ceftriaxone	2(1.5%)	3(2.3%)	125(96.2%)
Fourth generation:			
Cefepime	10(7.4%)	1(0.7%)	125(91.9%)
Carbapenems			
Meropenem	15(11%)	4(2.9%)	117(86%)
Aminoglycosides			
Amikacin	42(30.9%)		96(69.1%)
Gentamicin	33(24.3%)	5(3.7%)	98(72.1%)
Tobramycin	24(17.6%)	4(2.9%)	108(79.4%)
Fluoroquinolones			
Ciprofloxacin	7(5.1%)	4(2.9%)	125(91.9%)
Levofloxacin	11(8.1%)	2(1.5%)	123(90.4%)
Nitrofuran			
Nitrofurantoin	19(16.4%)	10(8.6%)	87(75%)
Folate inhibitors			
Trimethoprim/Sulfamethoxazole	23(17.7%)		107(82.3%)
Resistance patterns			
No	3(2.2%)		
MDR	18(13.2%)		
XDR	115(84.6%)		

Data were expressed as frequencies and percentages. MDR: multidrug-resistant. XDR: Extensively Drug-Resistant.

**Fig. 1:**
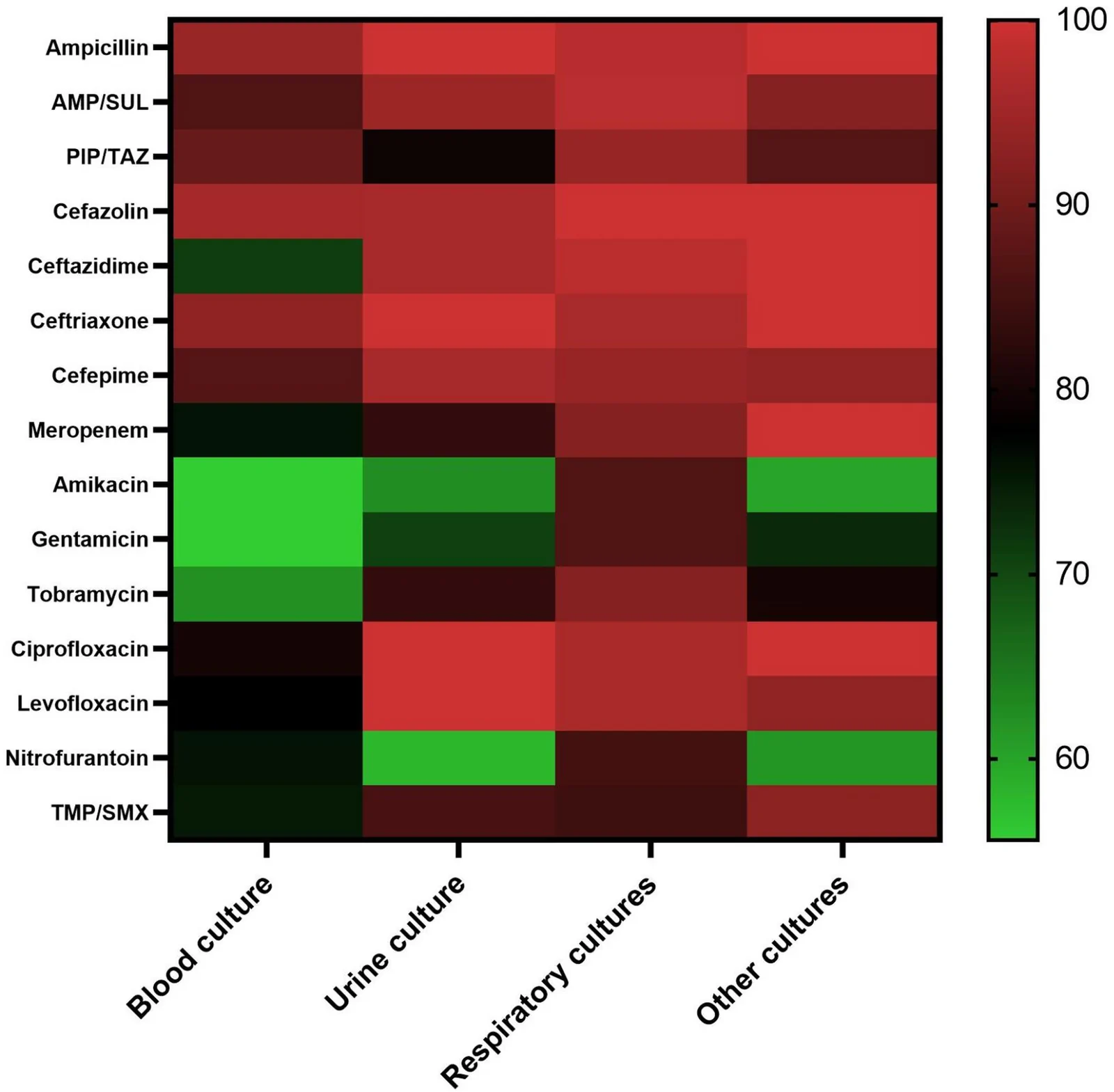
Heatmap of antibiotic resistance across different culture sources. PIP/TAZ: Piperacillin/Tazobactam. TMP/SMX: Trimethoprim/Sulfamethoxazole.

Based on the site of infection (Fig. 1), for bloodstream infection, cefazolin exhibited the highest resistance (95.6%), followed by ampicillin (94.1%), while amikacin and gentamicin exhibited the lowest resistance (55.6%). Bacterial species isolated from urine samples exhibited complete resistance to ampicillin, ceftriaxone, and fluoroquinolones, but they exhibited lower resistance to nitrofurantoin. Isolates causing respiratory infections exhibited high resistance to all antibiotics; they exhibited complete resistance to cefazolin (100%), followed by ceftazidime (98.1%) and Ampicillin/Sulbactam. Bacterial species from other cultures exhibited complete resistance to many antibiotics (ampicillin, cefazolin, ceftazidime, ceftriaxone, and meropenem). Out of 136 isolates, 115 (84.6%) were XDR, and 18 (13.2%) were MDR.

### Antimicrobial susceptibility and resistance patterns of the most common species

Table 5 compares the antimicrobial susceptibility of *K. pneumoniae* and *E. coli*. A significantly higher proportion of *K. pneumoniae* was resistant to ampicillin/sulbactam (*p* = 0.009), piperacillin/tazobactam (p < 0.001), and cefepime (*p* = 0.009) compared to *E. coli*, which had lower resistance than *K. pneumoniae*. *K. pneumoniae* also showed significant resistance to meropenem, aminoglycosides, and nitrofurantoin antibiotics (p<0.001), in comparison to *E. coli*. In Table 4, K. *pneumoniae* had significantly higher proportions of XDR (98.5%) than *E. coli*.

**Table 4:** Antibiotic susceptibility profile and resistance patterns between ESBL-producing *Klebsiella pneumoniae* and *Escherichia coli.*

Antibiotics	*Klebsiella pneumoniae* (*n* = 66)			*Escherichia coli* (*n* = 22)			*P*-value
	S	I	*R*	S	I	*R*	
Ampicillin	0(0%)	0(0%)	66(100%)	1(4.5%)	0(0%)	21(95.5%)	0.25
AMP/SUL	0(0%)	0(0%)	66(100%)	2(9.1%)	0(0%)	19(86.4%)	**0.009**
PIP/TAZ	0(0%)	0(0%)	66(100%)	6(27.3%)	3(13.6%)	13(59.1%)	**<0.001**
Cefazolin	0(0%)	0(0%)	66(100%)	2(9.1%)	0(0%)	20(90.9%)	0.06
Ceftazidime	1(1.5%)	4(6.1%)	61(92.4%)	1(4.5%)	1(4.5%)	20(91%)	0.692
Ceftriaxone	0(0%)	0(0%)	66(100%)	0(0%)	0(0%)	22(100%)	.
Cefepime	0(0%)	0(0%)	66(100%)	2(9.1%)	1(4.5%)	19(86.4%)	**0.009**
Meropenem	0(0%)	1(1.5%)	65(98.5%)	7(31.8%)	1(4.5%)	14(63.6%)	**<0.001**
Amikacin	9(13.6%)	0(0%)	57(86.4%)	18(81.8%)	0(0%)	4(18.2%)	**<0.001**
Gentamicin	10(15.2%)	3(4.5%)	53(80.3%)	15(54.5%)	2(9.1%)	8(36.4%)	**<0.001**
Tobramycin	6(9.1%)	1(1.5%)	59(89.4%)	8(36.4%)	13(13.6%)	11(50%)	**<0.001**
Ciprofloxacin	0(0%)	0(0%)	66(100%)	1(4.5%)	0(0%)	20(95.5%)	0.25
Levofloxacin	1(1.5%)	0(0%)	65(98.5%)	2(9.1%)	0(0%)	20(90.9%)	0.153
Nitrofurantoin	1(1.5%)	1(1.5%)	64(97%)	14(63.6%)	4(18.2%)	4(18.2%)	**<0.001**
TMP/SMX	7(10.6%)	0(0%)	59(89.4%)	4(18.2%)	0(0%)	18(81.8%)	0.456
Resistance patterns							
No	0(0%)			1(4.5%)			**<0.001**
MDR	1(1.5%)			7(31.8%)			
XDR	65(98.5%)			14(63.6%)			

Data were expressed as frequencies and percentages. P-value was significant if < 0.05. AMP/SUL: Ampicillin/Sulbactam. PIP/TAZ: Piperacillin/Tazobactam. TMP/SMX: Trimethoprim/Sulfamethoxazole. MDR: multidrug-resistant. XDR: Extensively Drug-Resistant.

**Table 5:** Distribution of antimicrobial resistance genes in all isolates and distribution difference between *Klebsiella pneumoniae* and *Escherichia coli*.

Gene families	Genes	All isolates (*N* = 136)	*Klebsiella pneumoniae* (*n* = 66)	*Escherichia coli* (*n* = 22)	*P*-value
β-lactamase gene families	TEM	128(94.1%)	63(95.5%)	20(90.9%)	0.595
	SHV	128(94.1%)	65(98.5%)	20(90.9%)	0.153
	CTX-M	110(80.9%)	64(97%)	17(77.3%)	**0.01**
ESBL-associated variants	CTX-M15	81(59.6%)	42(63.6%)	15(68.2%)	0.669
	SHV12	92(67.6%)	59(89.4%)	7(31.8%)	**<0.001**
	TEM63	83(61%)	47(71.2%)	11(50%)	0.069
Carbapenem gene	NDM1	107(78.7%)	57(86.4%)	15(68.2%)	0.056
Combinations	CTX-M&CTX-M15	77(56.6%)	42(63.6%)	12(54.5%)	0.448
	TEM&TEM63	83(61%)	47(71.2%)	11(50%)	0.069
	SHV&SHV12	92(67.6%)	59(89.4%)	7(31.8%)	**<0.001**
	NDM1&SHV&SHV12	78(57.4%)	52(78.8%)	5(22.7%)	**<0.001**
	NDM1&CTX-M&CTX-M15	72(52.9%)	42(63.6%)	8(36.4%)	**0.025**
	NDM1&TEM&TEM63	77(56.6%)	46(69.7%)	8(36.4%)	**0.005**
	CTX-M&CTX-M15& TEM&TEM63& SHV&SHV12	54(39.7%)	31(47%)	4(18.2%)	**0.017**
	CTX-M&CTX-M15& TEM&TEM63& SHV&SHV12&NDM1	52(38.2%)	31(47%)	3(13.6%)	**0.005**

Data were expressed as frequency (percentage). P-value was significant if < 0.05.

### β-lactamase, ESBL and carbapenem coding genes

**Fig. 2:**
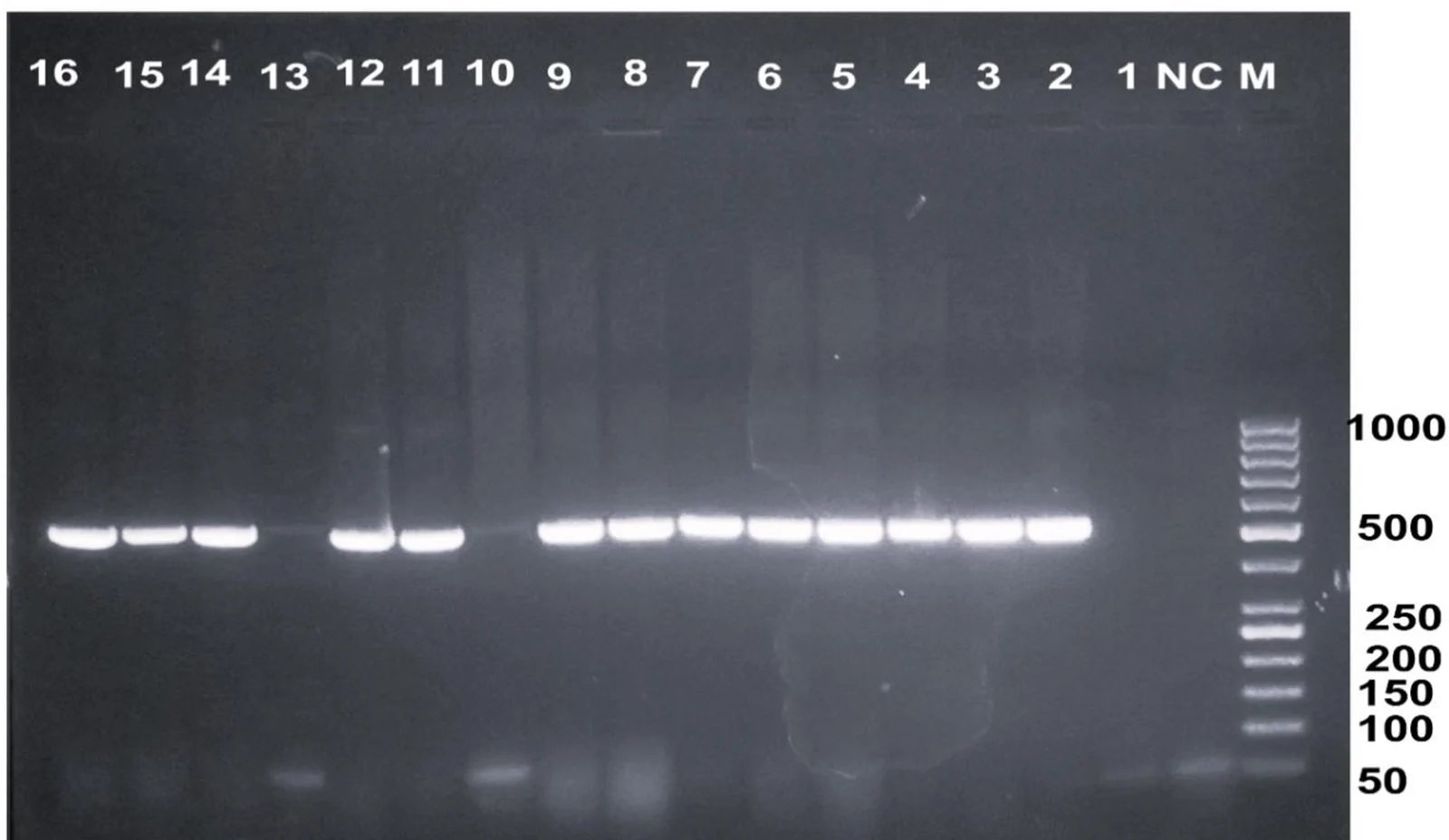
Agarose gel electrophoresis of the bla-CTX-M assayed using PCR. Lane 1, 50 bp DNA ladder; lane2, negative control; lanes 2–9, clinical isolates of CTX-M-positive *K. pneumoniae*; lanes 11–12 and 14–16, clinical isolates of CTX-M-positive *E. coli*; and lanes 10 and 13, clinical isolates of CTX-M-negative *K. pneumoniae* and *E. coli*.

Overall (Table 5), β-lactamase -coding bla_TEM_ and bla_SHV_ were the highest detected in isolates (94.1%), followed by bla_CTX−M_ (Fig. 2). SHV-12 was the most harbored among ESBL-coding variants (67.6%). 80.9%. 67.6% of isolates coharbored bla_SHV_ and _blaSHV−12_. Carbapenem-coding bla_NDM−1_ was harbored by 78.7% of isolates, and 52(38.2%) of isolates harbored all β-lactamase, ESBL, and carbapenem-coding genes.

Table 5 shows the differences in β-lactamase, ESBL, and carbapenem-encoding genes between *K. pneumoniae* and *E. coli*. A significantly higher proportion of *K. pneumoniae* harbored bla_CTX−M_ (*p* = 0.01) and bla_SHV−12_ (p<0.001) (Supplementary Figure S2) than *E. coli*, which produced a lower proportion of β-lactamase and ESBL genes than *K. pneumoniae*. *K. pneumoniae* significantly coharbored SHV and SHV-12 (p <0.001). In addition to *K. pneumoniae*, it significantly co-produced six β-lactamase and ESBL-coding genes compared to *E. coli* (*p* = 0.017). The two strains harbored a high proportion of other genes with no significant differences, such as bla_TEM 63_ (Supplementary Figure S3). Among *K. pneumoniae* and *E. coli*, 86.4% and 68.2%, respectively, harbored NDM1. *E. coli* (13.6%) showed a significant decrease in the harboring of all resistance genes compared to *K. pneumoniae* (47%).

### Outcomes of infection with ESBL-producing bacteria

Regarding the clinical outcomes of infection with ESBL (Supplementary Table S1), 69 (50.7%) patients with ESBL-producing bacteria died after infection. The mortality rate of patients with *K. pneumoniae* infection (56.1%) was significantly higher than that of patients with *E. coli* infection (27.3%).

### Risk factors associated with death in patients with ESBL-producing bacteria

In univariable analysis (Table 6), age was significantly and positively associated with death (OR = 1.026, CI: 1.009–1.043, *p* = 0.002). Patients who had a central line and urinary catheter were associated with death (OR = 3.011, CI: 1.158–7.832, *p* = 0.024, OR = 2.779, CI: 1.061–7.274, *p* = 0.037, consecutively). Patients who previously underwent surgical interventions and were admitted to the hospital within the last three months were significantly more likely to have a higher death rate. Patients with bloodstream and urinary tract infections showed a significant association with death. In addition, patients with *K. pneumoniae* infection showed a significant association with a higher mortality rate compared to *those with E. coli*. Furthermore, increasing relapses and hospital stays increased the risk of mortality. On the other hand, multivariable regression revealed that only age (*p* = 0.006), patients with relapses (*p* = 0.046), and hospital stay (OR = 1.093, CI: 1.028–1.162, *p* = 0.005) remained independently associated with mortality rate in infected patients with ESBL-producing bacteria. Notably, patients with ESBL-producing *K. pneumoniae* were marginally more associated with mortality than those with ESBL-producing *E. coli*.

**Table 6:** Univariable and multivariable logistic regression for death in ICU patients with ESBL-producing bacteria.

	Univariable				*P* value	Multivariable		
	*P* value	Odds Ratio	95% confidence intervals			Odds Ratio	95% confidence intervals	
			Lower	Upper			Lower	Upper
Age	**0.002**	1.026	1.009	1.043	**0.006**	1.053	1.015	1.093
Gender Male	0.309	0.704	0.358	1.384	-	-	-	-
Female	Ref.							
Malignancy type								
Malignancy Solid	0.399	1.355	0.669	2.743	-	-	-	-
Hematological malignancy	Ref.							
Culture source								
Blood	**0.024**	5	1.239	20.177	0.601	1.755	0.214	14.431
Urine	0.070	4	0.895	17.872	0.351	2.938	0.305	28.350
Respiratory	**0.021**	5.043	1.271	20.016	0.490	2.046	0.268	15.600
Others	Ref							
Blood CVC (yes)	**0.024**	3.011	1.158	7.832	-	-	-	-
Urinary catheter (yes)	**0.037**	2.779	1.061	7.274	-	-	-	-
Surgery (yes)	**0.001**	0.276	0.125	0.607	0.163	0.345	0.077	1.538
Prior antibiotic use (yes)	0.203	1.573	0.783	3.161	-	-	-	-
Past hospital admission (yes)	**0.011**	2.500	1.239	5.044	0.394	1.823	0.458	7.260
Relapses (yes)	**<0.001**	13.619	5.992	30.956	**0.046**	3.887	1.026	14.722
Comorbidity (yes)	0.790	1.105	0.528	2.313	-	-	-	-
Organism								
*Klebsiella pneumonia*	**0.023**	3.402	1.183	9.788	0.080	3.610	0.857	15.200
*Escherichia coli*	Ref.				Ref.			
Hospital days	**<0.001**	1.050	1.020	1.081	**0.005**	1.093	1.028	1.162

P-value was significant if < 0.05. Factors were not included in the multivariable regression due to multicollinearity based on the collinearity diagnostic test. CVC: central venous catheter.

## Discussion

This study comprehensively examined the distribution of ESBL-producing bacteria in ICU cancer patients, resistance patterns, β-lactamase, ESBL, and carbapenem genes, resistance patterns between *E. coli* and *Klebsiella pneumoniae*, and predictors of mortality in patients infected with ESBL-producing bacteria. Our results revealed that *K. pneumoniae* was the most common ESBL-producing bacterium in ICU cancer patients, followed by *E. coli* and Acinetobacter *baumannii*. The isolated bacteria showed high resistance to all cephalosporins and to meropenem. *E. coli* showed significantly lower resistance to β-lactam antibiotic combinations, cefepime, and meropenem than *K. pneumoniae* did. The most common β-lactamase genes in the isolates were bla_TEM_ and bla_SHV_. bla_SHV−12_ and blaTEM-63 were the most prevalent ESBL genes in the isolates. *K. pneumoniae* harbored β-lactamase, ESBL, and carbapenem genes significantly more than *E. coli* did. Most isolates were XDR (84.6%). Age, relapse status, and prolonged hospital stay remained predictors associated with mortality in patients with cancer infected with ESBL-producing bacteria. These results suggest that antibiotic stewardship programs should be improved, molecular surveillance of resistance genes should be strengthened, and high-risk patients should be quickly identified using authorized prognostic models.

The current study reveals that the main types of infection in ICU cancer patients were respiratory infection, followed by blood infection, and finally urinary tract infection. These results are contrary to a study performed in western China, which reported that urinary tract infections were the main type of nosocomial infection, followed by respiratory infections, and blood infections last^16^. This difference is due to our study focusing on ICU patients, most of whom were on mechanical ventilation and had infection prevention practices.

ESBL-producing bacteria resist β-lactam antibiotics; therefore, they are of clinical and epidemiological importance^17^. Of the isolated Gram-negative bacteria, 95.1% showed an ESBL-producing phenotype. Two consistent studies reported that 87% and 86.8% of the isolates were ESBL-producing^6,8^. Another study reported that 72.8% of isolates were ESBL-producing^16^. In contrast, the ESBL phenotype was detected in only 8% and 26.7% of isolates in Swiss ICUs and the Ankara Bilkent City hospital, respectively^18,19^. This high prevalence may be attributed to antibiotic misuse, transmission risks via invasive procedures, plasmid-mediated resistance, and recurrent infections.

*Klebsiella pneumoniae* was the most prevalent bacterium in our study, followed by *E. coli* and *Acinetobacter baumannii.* Unlike our study, a previous study reported *that E. coli* was the most common, followed by *K. pneumoniae*^20^. In addition, incompatible results were reported, with *E. coli* at the top, followed by *K. pneumoniae*, and *Acinetobacter baumannii* at the last^21^. The current study revealed that *Klebsiella pneumoniae* was the most responsible pathogen for blood and respiratory tract infections, followed by *E. coli*, which was found to be the primary cause of urinary tract infections, followed by *K. pneumoniae.* A result from a previous study is compatible with our result, which showed that *K. pneumoniae* and *E. coli* were the most dominant in blood culture^9^. In addition, Diyana et al. reported that *E. coli* was the most frequent cause of urinary tract infection, followed by *K. pneumoniae*, which is compatible with our study^22^. A study at Jimma University Medical Center reported that *Klebsiella ssp.* and *E. coli* were the most common causes of bloodstream infection, a finding that matches our results^23^.

Our analysis demonstrated isolates highly resistant to all antibiotics; the most concerning are cefazolin, ceftriaxone, and meropenem. Two studies by Jiang et al. and Kiya et al. disagree with our study, stating that the isolates showed high sensitivity to amikacin and meropenem^16,23^. The findings of low susceptibility to antibiotics in our study were due to high consumption of traditional antibiotics and recurrent infections, and consequently to immunosuppression. A more recent study on cancer patients stated that bacterial isolates showed high resistance to the cephalosporin group and high sensitivity to meropenem, which disagrees with our findings^24^. The use of carbapenems as a first-line therapy for ESBL-producing bacteria has contributed to selective pressure, driving the global spread and the emergence of carbapenem resistance, often through the production of carbapenemase enzymes^25^.

Our study showed that *K. pneumoniae* and *E. coli* displayed high resistance to most antibiotics, but *K. pneumoniae* showed significantly greater resistance than *E. coli* to certain antibiotic classes, involving penicillin combinations, aminoglycosides, cefepime, and carbapenems. A previous study in Ethiopia reported that *E. coli* and *K. pneumoniae* showed high resistance to ceftriaxone and cefepime, a finding that matches our study, but they found that they expressed high susceptibility to meropenem and amikacin, which is incompatible with our study^6^. Another inconsistent study reported that *E. coli* and *K. pneumoniae* had moderate resistance to ceftriaxone, ceftazidime, and cefazolin and low resistance to sulfamethoxazole-trimethoprim^22^. A study from Egypt demonstrated that *K. pneumoniae* showed complete resistance to most antibiotics, which agrees with our study^26^.

In clinical settings, multidrug-resistant bacteria increase because of increasing resistance to traditional antibiotics^6^. In our findings, most isolates were extensively drug-resistant, and others were multidrug-resistant isolates. Out of isolates, 84.6% were XDR and 13.2% were MDR, which differs from another study, which reported that 57% of isolates were XDR^20^. The spread of XDR affects patient outcomes and reduces therapeutic options. A previous study reported that 32.2% of isolates were XDR^27^.

Multidrug-resistant Enterobacteriaceae pose a significant warning in the treatment of urinary tract infections^22^. Our findings revealed that most *K. pneumoniae* were XDR compared to *E. coli*. However, an equal percentage of XDR *K. pneumoniae* and *E. coli* was present in a different study^20^. Arsho Advanced Medical Laboratory stated that 42.2% and 35.7% of *E. coli* strains were MDR and XDR, respectively, and 37.5% of *K. pneumoniae* were XDR^28^. However, 63.6% of the isolated *E. coli* in our study were XDR, and 98.5% of the isolated *K. pneumoniae* were XDR. This is due to the immunosuppression status of ICU cancer patients in our study and the frequent misuse of antibiotics. MDR-GNB frequently demonstrate both ESBL and carbapenem-resistant phenotypes, complicating treatment options, particularly in high-risk patients such as those with cancer^29^.

Regarding molecular detection of β-lactamase genes, bla_TEM_ and bla_SHV_ were the most common, followed by bla_CTX−M_. Around 40% of isolates harbored all β-lactamase and ESBL genes. But in reverse, in Uganda and Iran, the most common was bla_CTX−M_, followed by bla_TEM_; bla_SHV_ was the least identified^20,24^.

*K. pneumoniae* and *E. coli* expressed high percentages of ESBL genes, but *K. pneumoniae* differed significantly, as it harbored bla_CTX−M_, bla_SHV12_, and a significant number of *K. pneumoniae* isolates harbored all genes. In *K. pneumoniae*, SHV was the most common β-lactamase gene, followed by CTX-M and TEM, but in *E. coli*, TEM and SHV were the most frequent β-lactamase genes, followed by CTX-M. Two dissimilar studies reported that CTX-M was the most prevalent β-lactamase gene in *E. coli*, followed by TEM and SHV^24,30^. In our study, 89.4% of *K. pneumoniae* harbored bla_SHV12_. SHV-12 is identified as an ESBL gene, detected across all continents, and is broadly significant within the *Enterobacterales*, which includes *K. pneumoniae*, underlining its global epidemiological importance^31^. It contributes to its multidrug resistance phenotype, making infections caused by it challenging to treat with conventional β-lactam antibiotics and necessitating alternative therapeutic strategies^32^.

The spread of ESBL genes is significantly facilitated by mobile genetic elements, such as plasmids and transposons, which can carry multiple resistance genes and contribute to their dissemination^25^. Our findings showed the coexistence of two or more genes in the isolates. Another Egyptian study reported that the coexistence of CTX-M and CTX-M15 was lower than that in our study, and the coexistence of CTX-M, SHV, and TEM was lower than that in our study^33^. *E. coli* isolates frequently harbor multiple β-lactamase genes, with bla_CTX−M_, bla_TEM_, and bla_OXA−1_ being commonly detected, often contributing to multidrug resistance phenotypes^34^.

In our results, 63.6% and 68.2% of *K. pneumoniae and E. coli*, respectively, produced CTXM-15. CTX-M-15 is the most predominant ESBL type worldwide, having displaced other ESBL variants and is a major contributor to resistance in GNB^35^. CTX-M-15-producing *E. coli* and *K. pneumoniae* are a significant cause of resistance to ESBL, posing a substantial global public health concern, the premier driver of resistance to ESBL (such as cefotaxime, ceftriaxone, and ceftazidime) in *Enterobacterales*^36^. The widespread dissemination of CTX-M-15 is largely facilitated by its location on mobile genetic elements such as plasmids, insertion sequences, and transposons, enabling efficient horizontal gene transfer among bacterial species^25^. TEM-63 was identified in three genera of South African isolates: *K. pneumoniae*, *E. coli*, and *Proteus mirabilis*^37^. In our findings, 61% of bacteria harbored TEM63. Variants of TEM enzymes, such as TEM-63, have progressed through mutations to acquire extended-spectrum activity, enabling them to hydrolyze oxyimino-cephalosporins such as ceftazidime and cefotaxime^38^.

ESBL-producing *Enterobacterales* often exhibit co-resistance to multiple oral antibiotics, referring to the presence of additional resistance genes beyond ESBLs^39^. In our study, the isolates exhibited co-resistance to β-lactam, ESBL, and meropenem antibiotics. Meropenem-resistant *Enterobacterales* frequently carry multiple β-lactamase types, including KPC or OXA-48-like carbapenemases, in combination with ESBLs and/or AmpC β-lactamases^40^. Carbapenem resistance in *Enterobacterales* is increasing worldwide, and it has global concern due to limited treatment options^41^. Hematological malignancy patients infected with carbapenem-resistant bacteria have a high death rate owing to infection complications^42^. The bla_NDM_ gene is an important contributor to carbapenem resistance^43^. Our findings demonstrated that 78.8% of isolates harbored NDM1 (carbapenem determinant), and 38.2% of isolates harbored all β-lactamase, ESBL, and carbapenem genes. 86.4% of isolated *K. pneumoniae* produced NDM1. Consistent with our study, 82% of *K. pneumoniae* isolated from pediatric hospitals harbored NDM^43^. A study performed on bladder cancer patients demonstrated that 32% of isolates expressed NDM as a carbapenem determinant^26^. In addition, a study on pediatric cancer patients demonstrated that 67% of bacterial isolates produced NDM that confers resistance to carbapenem^44^.

A previous study proved that admission of patients to the ICU is considered one of the risk factors of death^45^. Mortality in neutropenic patients after chemotherapy is mainly associated with bacterial infections^3^. Hematological malignancy patients are extremely susceptible to blood infection by GNB because of immunosuppression, intensive treatment, and frequent invasive procedures, which is the main cause of mortality^9^. In our study, about fifty patients died. Jiang et al. reported that only 10.9% of patients died^16^. This difference is due to most of our patients having respiratory infections, whereas in another study, most patients had urinary tract infections. In univariate regression, age, hospital admission duration, previous hospital admission, blood and respiratory infection, previous surgery, urinary catheter, blood CVC, relapses, and infection with *K. pneumoniae* were predictors of mortality in ICU cancer patients. In a previous study, surgery, urinary catheter, urinary, blood, and respiratory infections, and hospital admission duration were predictors of death^16^. Findings that agree with our results. Patient age, relapsed patients, and prolonged hospital admission are independently associated with death in patients with ESBL-producing bacteria in multivariate regression. A study in Mexico, compatible with our results, reported that relapses are independently associated with mortality in cancer patients^46^. Also, research on the US population stated that age is one of the risk factors associated with mortality in patients with malignancy^47^. An inconsistent study from China reported that only male patients (comparable to female patients) and patients receiving chemotherapy were independently associated with mortality in patients with malignancy and COVID-19^48^.

ESBL-producing bacteria pose a significant challenge to patients with cancer in the ICU because they are associated with MDR and XDR patterns. The rise of antimicrobial resistance is underscored by the dominance of β-lactamase gene families (TEM, SHV, CTX-M), ESBL genes (SHV-12, TEM-63, CTX-M-15), and the carbapenemase gene NDM1. Our results underscore the importance of early detection, antibiotic stewardship, and infection control to improve patient outcomes. Patient age, relapse rate, and prolonged hospital admission are associated with mortality in patients with cancer in the ICU. Our study has some limitations: It was conducted in a single center, and anaerobic bacterial cultures and identification were not performed because specialized facilities and equipment were not available. We did not investigate other ESBL genes, additional carbapenemase genes, or non-enzymatic mechanisms (e.g., porin loss and efflux pump overexpression). However, our study provides significant insights into the prevalence of ESBL-producing bacteria, their resistance patterns, and associated resistance mechanisms among patients with cancer in the ICU, as well as predictors of mortality in patients with cancer in the ICU with ESBL-producing bacteria.

## Conclusion

Our study demonstrates the high prevalence of β-lactamase-producing bacteria in ICU cancer patients. *K. pneumoniae* was the most common bacterium, followed by *E. coli*. The strains demonstrated high resistance to all antibiotics. *K. pneumoniae* showed complete resistance to many antibiotics. *E. coli* showed high resistance to most antibiotics. TEM and SHV were the main β-lactamase genes harbored by the isolates. Isolates showed significant resistance to meropenem (carbapenem, the last-line treatment for infections resistant to cephalosporins). A high percentage of isolates harbored more than one gene. XDR isolates were prevalent in patients. Age, hospital stay, and relapses are the main predictors of mortality in the studied patients. These findings imply that we should enhance molecular surveillance of resistance genes, boost antibiotic stewardship, and infection control programs.

## Materials and methods

### Study design and setting

This cross-sectional study was conducted at the South Egypt Cancer Institute, Assiut University, from March 2025 to December 2025. All cancer patients in the intensive care unit with apparent symptoms of infection were included in the study.

### Data collection (demographic, laboratory, and clinical data)

Demographic and clinical data were collected for each patient included in the study. Demographic variables included sex and age. Clinical variables included diagnosis, treatment, use of urinary catheterization, mechanical ventilation, and other invasive procedures during hospitalization. Past surgery in the last 3 months and past hospital and ICU admissions were inquired about. In addition to comorbidities, hospital stay time, relapse status of patients, and past antibiotic use were also inquired about. These data were collected from hospital medical records and used to assess potential risk factors associated with bacterial infections.

### Samples collection

Samples (blood, urine, stool, surgical, and respiratory cultures) were collected for routine cultures at the Microbiology Laboratory, South Egypt Cancer Institute, under complete aseptic conditions. Cultures were processed according to the laboratory’s standard operating procedure (SOP) of the microbiology unit (Clinical and Laboratory Standards Institute (CLSI) guided).

### Bacteriological culture and identification

Blood samples were processed using the Render blood culture device (Model BC32, SN: 19070072c) according to the manufacturer’s instructions. Blood samples that show colorimetric changes, urine, stool, surgical, and respiratory samples were screened on nutrient, MacConkey, 5% sheep blood agar, chocolate, Cystine-lactose-electrolyte-deficient (CLED), Xylose Lysine Deoxycholate (XLD), and Mannitol salt agar (Oxoid™, Thermo Fisher Scientific, UK) as appropriate for each culture at 37 °C for 18–24 h. Pure cultures were identified based on Gram staining, colony morphology on agar plates, and using a VITEK 2 compact (bioMérieux, Inc., USA, SN: VK2C14625).

### Susceptibility test

In accordance with the Clinical Laboratory Standards Institute (CLSI), antibiotic resistance profile tests and minimum inhibitory concentration (MIC) determination for clinical isolates were performed using the VITEK 2 Compact15 (bioMérieux, Inc., USA, SN: VK2C14625). VITEK 2 antimicrobial susceptibility testing cards (AST cards), specific for Gram-negative bacteria, were used according to the manufacturer’s instructions. The susceptibilities of Gram-negative bacteria were tested using the following fifteen antibiotics: penicillin (ampicillin, ampicillin/sulbactam, piperacillin/tazobactam), cephalosporins (cefazolin, ceftazidime, ceftriaxone, cefepime), carbapenem (meropenem), aminoglycosides (amikacin, gentamicin, tobramycin), fluoroquinolones (ciprofloxacin, levofloxacin), nitrofurans (nitrofurantoin), and folate inhibitors (trimethoprim/sulfamethoxazole).

An isolate resistant to at least one agent in three antimicrobial categories is considered MDR. An isolate being non-susceptible to at least one agent in all but two or fewer antimicrobial categories is considered XDR^14^.

### Combined disk test for phenotypic detection of ESBL

Combination disk test (CDT), as recommended by the CLSI, was performed on all Gram-negative bacterial isolates presumed to be ESBL producers. Presumptive isolates from initial screening were emulsified in 4– 6 ml of peptone water to adjust the inoculum density to that of the 0.5 McFarland turbidity standard. In this test, the cefotaxime (30 µg) disk alone and in combination with clavulanic acid (cefotaxime + clavulanic acid, 30/10 µg) were applied onto a plate of Mueller–Hinton agar (MHA), which was inoculated with the test strain and then incubated for 16–18 h at 35 ± 2 °C in ambient air. The bacterial isolate showing an increase of ≥ 5 mm in the inhibition zone of the combination disk compared to the cefotaxime disk alone was considered an ESBL producer according to CLSI guidelines (2025)^49^.

### Detection of β-lactamase, ESBL and carbapenem genes

For bacterial genetic material extraction, colonies from an overnight bacterial culture were transferred into 100 µL of sterile distilled water and placed in a preheated ARKITIK thermal cycler (Thermo Scientific, USA, SN: AKC481205729) at 95 °C for 10 min to lyse the bacterial cells, then centrifuged at 12,000–14,000 rpm for 5 min. The supernatant containing crude bacterial DNA was then stored at − 80 °C until further use^50^.

The collected DNA samples were amplified using the ARKTIK thermal cycler to detect resistance genes^30,33,37,51–54^(Supplementary Table S2), including β-lactamase gene families (TEM, SHV, CTX-M), ESBL-associated variants (TEM63, SHV12, CTX-M-15), and the carbapenem resistance gene (NDM1). Conventional individual PCR assays were performed for each of the seven target resistance genes using gene-specific primer pairs. Each gene was amplified in a separate PCR reaction. The 25-µL PCR amplification mixture contained 0.2 µM of each primer concentration, 2–3 µL of DNA, and 12.5 µL of Thermo Scientific DreamTaq Green PCR Master Mix (2X) (USA). The cycling conditions were as follows: an initial denaturation at 95 °C for 3 min, followed by 35 cycles consisting of 95 °C for 30 s, annealing temperatures (55–59) °C (depending on the target gene) for 30 s, and 72 °C for 1 min, followed by a final extension at 72 °C for 10 min.

PCR products along with the Thermo Scientific GeneRuler 50–100 bp DNA Ladder were run on a 2% agarose gel and separated by electrophoresis in 1x TAE buffer. PCR products were visualized with ethidium bromide under UV light using a TRZol UV transilluminator, Model: Digidoc 12, USA.

### Statistical analysis

Data were collected and analyzed using SPSS version 27. Quantitative data were expressed as the median and the range (minimum-maximum). Nominal data were presented as numbers (n) and percentages (%). For comparison of categorical data, the chi-square (χ^2^) test was performed. Univariable and multivariable logistic regression analyses were performed to determine the predictive factors for death among patients with ESBL-producing bacteria. A *P-value* was considered significant if it was < 0.05.

## Supplementary Information

Below is the link to the electronic supplementary material.

## Data Availability

All data supporting the findings of this study are available within the paper and its Supplementary Information.
