# Supplementary material for: Extensively drug-resistant ESBL-producing enterobacterales and coexisting antimicrobial resistance genes are associated with mortality in critically ill patients with cancer
Source: Sci Rep. 2026 Sep 2;16:27530. doi: 10.1038/s41598-026-67637-0 (PMC13538596; doi:10.1038/s41598-026-67637-0)
Supplement: Supplementary file 1 [file 41598_2026_67637_MOESM1_ESM.docx]

**Supplementary tables**

**Supplementary Table S1:** Outcomes of infection with ESBL-producing bacteria and outcome difference between *Klebsiella pneumoniae* and *Escherichia coli* in the ICU.

|  |  | All isolates  (N=136) | *Klebsiella pneumoniae* (n=66) | *Escherichia coli* (n=22) | P-value |
| --- | --- | --- | --- | --- | --- |
| Death | No | 67(49.3%) | 29(43.9%) | 16(72.7%) | **0.019** |
|  | Yes | 69(50.7%) | 37(56.1%) | 6(27.3%) |  |

Data were expressed as frequency (percentage). P-value was significant if < 0.05.

**Supplementary Table S2:** PCR primers for the detection of ESBL genes

| Primers | Sequences 5´→3´ | Size (bp) | Reference |
| --- | --- | --- | --- |
| TEM | **F: TTGGGTGCACGAGTGGGTTA** | **504** | **^52^** |
|  | **R: TAATTGTTGCCGGGAAGCTA** |  |  |
| TEM63 | **F: TCGGGGAAATGTGCG** | **992** | **^37^** |
|  | **R: CTTGGTCTGACAGTTACC** |  |  |
| SHV | **F: CTTTATCGGCCCTCACTCAA** | **237** | **^53^** |
|  | **R: AGGTGCTCATCATGGGAAAG** |  |  |
| SHV12 | **F: GGTTATGCGTTATATTCGCC** | **865** | **^51^** |
|  | **R: TTAGCGTTGCCAGTGCTC** |  |  |
| CTX-M | **F: GAAGGTCATCAAGAAGGTGCG** | **560** | **^30^** |
|  | **R: GCATTGCCACGCTTTTCATAG** |  |  |
| CTX-M15 | **F: CACACGTGGAATTTAGGGACT** | **996** | **^33^** |
|  | **R: GCCGTCTAAGGCCATAAACA** |  |  |
| NDM1 | **F: GGTTTGGCGATCTGGTTTTC** | **621** | **^54^** |
|  | **R: CGGAATGGCTCATCACGATC** |  |  |

**Figures**

**Supplementary Figure S1:** The pie chart shows the distribution of infection sources.

**Supplementary Figure S2:** Agarose gel electrophoresis of the bla-SHV12 assayed using PCR. Lane 1, 100bp DNA ladder; lanes 1-3 and lane 5, clinical isolates of SHV12-positive *E. coli*; lanes 7-15, clinical isolates of SHV12-positive *K. pneumoniae*.

**Supplementary Figure S3:** Agarose gel electrophoresis of the bla-TEM63 assayed using PCR. Lane 1, 100bp DNA ladder; lanes 1-3, clinical isolates of TEM63-positive *E. coli*; lanes 7, 10, 16, and lanes 13-14, clinical isolates of TEM63-positive *K. pneumoniae*.
